# Serum zinc, selenium, and vitamin D levels in patients with acne vulgaris: A case–control study

**DOI:** 10.1111/jocd.16494

**Published:** 2024-07-25

**Authors:** Armaghan Kazeminejad, Zohreh Hajheydari, Seyed Shokoufe Taghian, Nasim Gholizadeh

**Affiliations:** ^1^ Department of Dermatology Faculty of Medicine, Mazandaran University of Medical Sciences Sari Iran; ^2^ Student Research Committee, Faculty of Medicine, Mazandaran University of Medical Sciences Sari Iran

**Keywords:** acne vulgaris, case–control, selenium, vitamin D, zinc

## Abstract

**Background:**

Acne vulgaris is a very common inflammatory skin disease that significantly impacts the quality of life of affected individuals. Previous studies have indicated that individuals with acne vulgaris often have low levels of zinc, selenium, and vitamin D. However, these three nutrients have rarely been collectively examined in a single study. The objective of this study was to compare serum levels of zinc, selenium, and vitamin D in patients with acne vulgaris in comparison to a control group.

**Methods:**

This case–control study included 100 adult patients with acne vulgaris attending a dermatology clinic, in 2020. A group of 100 patients without acne vulgaris attending the same clinics were evaluated as controls. Participants' sociodemographic characteristics, including age, sex, occupation, alcohol consumption, and tobacco smoking were recorded. In addition, anthropometric indices, such as weight and height, were measured and the body mass index (BMI) was calculated accordingly. Acne severity was determined using Tutakne and Chari's grading system. Serum zinc, selenium, and vitamin D levels were evaluated in peripheral blood samples of all the participants.

**Results:**

Acne patients and controls were comparable regarding age, sex, weight, height, BMI, occupation, alcohol consumption, and tobacco smoking (*p* > 0.05). Serum zinc, selenium, and vitamin D levels were all significantly lower in acne patients than in controls (*p* < 0.001). Furthermore, the frequency of abnormal zinc and vitamin D levels was significantly higher in acne patients (*p* = 0.002 and *p* = 0.001, respectively), but there was no significant difference between the two groups regarding abnormal serum selenium levels (*p* = 0.228). Also, serum zinc, selenium, and vitamin D levels lower levels in patients with grade 4 acne compared to other disease severity grades (*p* < 0.001).

**Conclusions:**

Patients with acne vulgaris have lower levels of serum zinc, selenium, and vitamin D compared to the control group. Additionally, there is a correlation between the severity of the disease and lower levels of these nutrients.

## 1 INTRODUCTION

Acne vulgaris is a prevalent inflammatory dermatological condition that affects the pilosebaceous units, impacting around 9% of the global population.^1^ It is a chronic condition that predominantly affects adolescents during puberty; nevertheless, adult‐onset acne can occur in older individuals.^2^ The incidence rates of acne vulgaris vary between males and females. Acne vulgaris primarily affects seborrheic zones, particularly those found on the face. However, it is also observed on the back and thoracic area.^3^ Acne has detrimental effects on individuals' quality of life and self‐esteem. Additionally, it is linked to heightened risks of physical scarring and psychological disorders, such as, anxiety, depression, and suicidal thoughts.^4^

The skin is the organ with the third highest zinc content, and zinc deficiency is associated with various skin diseases.^5^ A recent meta‐analysis has provided confirmation of low serum zinc levels in individuals with acne.^6^ Another review demonstrated the efficacy of zinc‐based products, both those containing zinc as the sole active ingredient and those with zinc as an adjunctive component, in treating acne.^7^

On the other hand, selenium levels have been linked to multiple skin diseases and their severity, with elevated selenium levels being associated with a protective effect in specific skin conditions.^8^ Selenium has been shown to regulate sebum production and possess anti‐inflammatory properties, suggesting its potential efficacy in the treatment of acne.^3^ Furthermore, low blood selenium levels have been observed in individuals with acne vulgaris.^8^

Vitamin D exerts a regulatory influence on the immune system. Moreover, it plays a role in the regulation of keratinocyte and sebocyte proliferation and differentiation. Vitamin D possesses antioxidant and anticomedogenic properties, and may play a role in the development of acne.^9^ Research has indicated a notable increase in the prevalence of vitamin D deficiency among individuals with acne vulgaris when compared to control groups.^10^

Due to the significant occurrence of acne vulgaris and its consequential physical and psychosocial effects, it is crucial to investigate the relationship between specific nutrients and the development of acne vulgaris to enhance existing acne treatments. Thus, the objective of this study was to assess the serum levels of zinc, selenium, and vitamin D in patients with acne vulgaris and compare them to a control group.

## 2 METHODS

### 2.1 Participants and study design

This case–control study included 100 patients with acne vulgaris attending a dermatology clinic, in 2020. A group of 100 patients without acne vulgaris attending the same clinics were evaluated as controls. The inclusion criteria for cases were age >18 years and the clinical diagnosis of acne vulgaris by the same dermatologist. The inclusion criterion for controls was age >18 years. The exclusion criteria were presence of other types of acne, such as acne mechanica, receiving oral anti‐acne treatments, vitamin D, zinc, or selenium supplements, or phototherapy within 3 months prior to the study, oral corticosteroid use, and chronic underlying diseases, including diabetes, hypertension, and the like.

The sample size was calculated as at least 92 in each group based on a type I error of 0.05, power of 95%, mean serum zinc level of 102.42 ± 18.1 μg/dL in controls and 95.67 ± 4.58 μg/dL in moderate acne cases of the study by Saleh et al.^11^ The study obtained ethics approval from the Ethics Committee and adheres to the principles outlined in the Declaration of Helsinki. All participants provided written informed consent.

Participants' sociodemographic characteristics, including age, sex, occupation, alcohol consumption, and tobacco smoking were recorded. In addition, anthropometric indices, such as weight and height, were measured using a standard digital scale and a nonstretchable tape, respectively. Subsequently, body mass index (BMI) was calculated for each participant by dividing weight (kg) by the square of height (m). Moreover, in patients with acne vulgaris, disease duration, distribution of acne, previous acne treatment and its type, as well as family history of acne were recorded. Acne severity was determined using Tutakne and Chari's grading system^12^:

- Grade 1: comedones, occasional papules;
- Grade 2: papules, comedones, few pustules^1^, ^2^, ^3^;
- Grade 3: predominant pustules (>3), nodules, abscesses; and
- Grade 4: mainly cysts, abscesses, widespread scarring.

Peripheral blood samples (5 mL) were obtained from each participant. Two milliliters of the sample were placed in a test tube containing anticoagulants, while the remaining 3 mL were placed in a separate test tube without anticoagulants. The 3‐mL sample was centrifuged at 2500 rpm for 15 min and subsequently divided into two microtubes. The samples were then stored at temperatures of −20°C and −80°C, respectively. Serum zinc and selenium levels were determined using atomic absorption spectroscopy, while serum vitamin D levels were measured using a commercially available kit from EUROIMMUN Co., France.

### 2.2 Data analysis

Data analysis was conducted using SPSS software (version 26.0, Armonk, NY: IBM Corp., USA). Continuous variables were summarized using measures of central tendency (mean) and dispersion (standard deviation). Categorical variables were described by calculating frequencies and percentages. Chi‐square test and the independent *t*‐test were used to compare categorical and continuous variables between cases and controls, respectively. The one‐way ANOVA test was applied to compare continuous variables by disease severity grades. *p*‐Values <0.05 were considered statistically significant.

## 3 RESULTS

Table 1 depicts the general characteristics of the study participants. Acne patients and controls were comparable regarding age, sex, weight, height, BMI, occupation, alcohol consumption, and tobacco smoking.

**TABLE 1:** General characteristics of the study participants.

Variables	Total (*n* = 200)	Acne vulgaris (*n* = 100)	Control (*n* = 100)	*p*‐Value^a^
Age (years), mean (SD)	25.4 (6.0)	25.5 (6.3)	25.3 (5.6)	0.795^b^
Sex, *N* (%)				
Male	58 (29.0)	31 (31.0)	27 (27.0)	0.533
Female	142 (71.0)	69 (69.0)	73 (73.0)	
Weight (kg), mean (SD)	65.1 (11.3)	65.0 (11.8)	65.3 (10.7)	0.846^b^
Height (cm), mean (SD)	167.2 (7.5)	166.5 (6.5)	168.0 (8.3)	0.159^b^
BMI (kg/m^2^), mean (SD)	23.2 (3.2)	23.3 (3.4)	23.1 (2.9)	0.547^b^
Occupation, *N* (%)				
Homemaker	44 (22.0)	22 (22.0)	22 (22.0)	0.972
University student	99 (49.5)	49 (49.0)	50 (50.0)	
Civil servant	50 (25.0)	26 (26.0)	24 (24.0)	
Self‐employed	7 (3.5)	3 (3.0)	4 (4.0)	
Alcohol consumption, *N* (%)	10 (5.0)	5 (5.0)	5 (5.0)	1.000
Tobacco smoking, *N* (%)	24 (12.0)	10 (10.0)	14 (14.0)	0.384

Abbreviation: BMI, body mass index.

^a^ Analyzed by chi‐square test.

^b^ Analyzed by the independent *t*‐test.

Disease‐related variables of acne patients are shown in Table 2. The mean disease duration was 59.9 ± 6.71 months. In terms of acne severity, the majority of patients were classified as having grade 3 (31%), followed by grade 2 (29%). Acnes were mostly present only on the face (41%). Eighty‐four patients (84%) had previously received treatments for acne vulgaris (earlier than 3 months ago), the most common of which was topical and oral treatments (46%). Moreover, 34 patients (34%) had a family history of acne.

**TABLE 2:** Disease characteristics of acne vulgaris patients.

Variables	Values
Duration (months), mean (SD)	59.9 (6.71)
Acne severity grade, mean (SD)	2.4 (1.0)
Acne severity grade, *N* (%)	
Grade 1	23 (23.0)
Grade 2	29 (29.0)
Grade 3	31 (31.0)
Grade 4	17 (17.0)
Distribution, *N* (%)	
Face	41 (41.0)
Face & trunk	34 (34.0)
Face & neck	4 (4.0)
Face, neck, and trunk	21 (21.0)
Previous acne treatment, *N* (%)	84 (84.0)
Type of previous treatment, *N* (%)	
Topical	31 (31.0)
Herbal	6 (6.0)
Oral	17 (17.0)
Topical & oral	46 (46.0)
Family history of acne, *N* (%)	34 (34.0)

**FIGURE 1:**
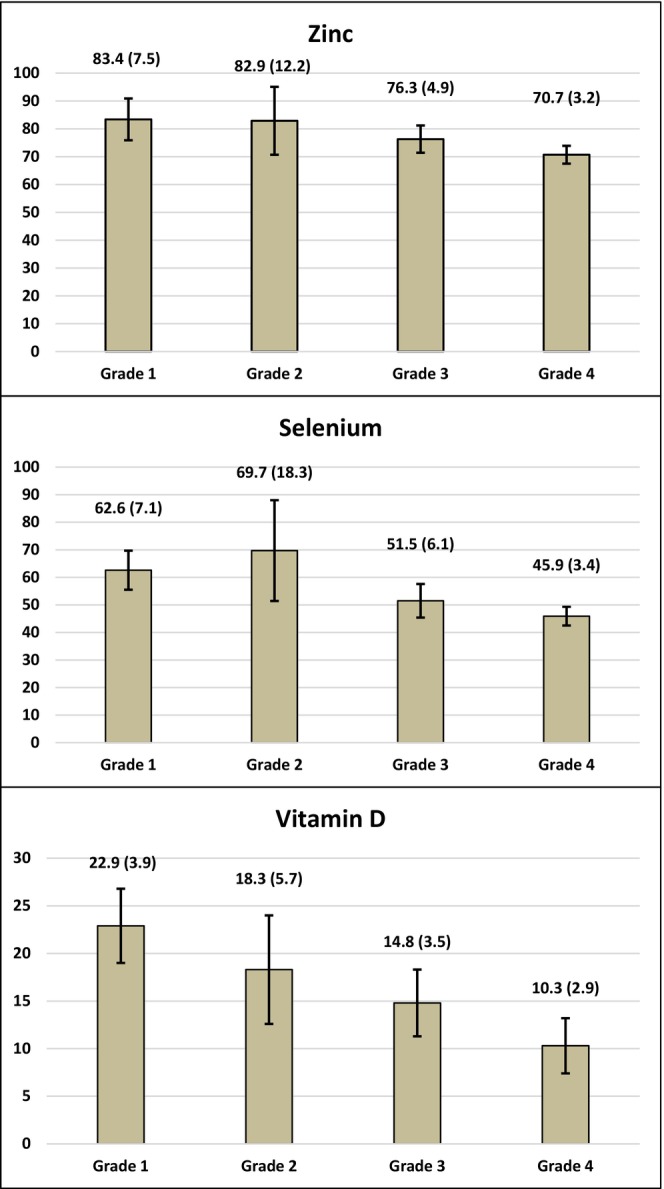
Mean (SD) of serum zinc (μg/dL), selenium (μg/L), and vitamin D (ng/mL) levels by acne vulgaris severity grade (error bars indicate standard deviation).

Serum zinc, selenium, and vitamin D levels were all significantly lower in acne patients than in controls (*p* < 0.001).(Table 3) Furthermore, the frequency of abnormal zinc and vitamin D levels was significantly higher in acne patients (*p* = 0.002 and *p* = 0.001, respectively). Nevertheless, there was no significant difference between the two groups regarding abnormal serum selenium levels (*p* = 0.228). Serum zinc, selenium, and vitamin D levels by disease severity grade in acne patients are demonstrated in Figure 1, which reveals lower levels in patients with grade 4 acne compared to other disease severity grades (*p* < 0.001).

**TABLE 3:** Serum zinc, selenium, and vitamin D levels.

Variables	Acne vulgaris (*n* = 100)	Control (*n* = 100)	*p*‐Value^a^	Effect size (95% CI)
Zinc (μg/dL), mean (SD)	78.9 (9.3)	105.5 (19.1)	<0.001	−1.77 (−2.10; −1.44)^c^
Selenium (μg/L), mean (SD)	58.3 (14.2)	100.6 (29.6)	<0.001	−1.82 (−2.15; −1.49)^c^
Vitamin D (ng/mL), mean (SD)	16.9 (6.0)	27.7 (12.5)	<0.001	−1.10 (−1.40; −0.80)^c^
Zinc, *N* (%)				
Abnormal	12 (12.0)	1 (1.0)	0.002^b^	13.5 (1.91; 582.69)^d^
Normal	88 (88.0)	99 (99.0)		
Selenium, *N* (%)				
Abnormal	12 (12.0)	7 (7.0)	0.228^b^	1.81 (0.62; 5.68)^d^
Normal	88 (88.0)	93 (93.0)		
Vitamin D, *N* (%)				
Abnormal	86 (86.0)	65 (65.0)	0.001^b^	3. 31 (1.57; 7.19)^d^
Normal	14 (14.0)	35 (35.0)		

^a^ Analyzed by the independent *t*‐test.

^b^ Analyzed by the Chi‐squared test.

^c^ Standardized mean difference by Cohen's d.

^d^ Odds ratio.

## 4 DISCUSSION

The current study revealed significantly lower serum zinc, selenium, and vitamin D levels in patients with acne vulgaris compared to controls. Moreover, lower levels of these nutrients were associated with more severe acne, with the lowest levels observed in grade 4.

Zinc is an essential trace element that plays a crucial role in the composition of proteins and other biological molecules. It serves multiple biological functions such as acting as an antioxidant, maintaining the integrity of the skin and mucous membranes, and promoting various enzymatic and transcriptional responses.^13^ Zinc deficiency enhances the conversion of testosterone to dihydrotestosterone, which in turn stimulates sebum production.^11^, ^14^ Moreover, zinc deficiency can cause abnormalities including pathogen infection, immune dysregulation, and activation of inflammatory genes.^15^

Consistent with our findings, Usmani et al.^16^ reported significantly lower serum zinc levels in patients with acne vulgaris compared to controls. Likewise, in their systematic review and meta‐analysis on 12 studies published from 1977 to 2016 including 690 acne patients and 594 controls, Yee et al.^6^ showed significantly lower serum zinc levels in acne patients. They also demonstrated the efficacy of zinc supplementation for the treatment of acne, especially lowering the number of inflammatory papules. On the other hand, Sultana et al.^17^ reported slightly lower serum zinc levels in acne patients than in controls, but without statistical significance. Similar results were demonstrated by Chandra, showing slightly but not significantly lower serum zinc levels in acne patients; nonetheless, in harmony with the present study, they found a significant association between acne severity and serum zinc levels.^18^

There are multiple possible explanations for the disparity between our findings and those of prior research. The participants in the current study had not taken any zinc supplements in the 3 months leading up to the study; however, the participants' nutrition status was unclear in terms of their consumption of foods containing zinc. This might have affected the results. Of note, the sampling time is a crucial factor that may account for the variation in serum zinc levels among acne vulgaris patients in different studies. This is because the zinc status can fluctuate throughout the day due to diurnal variation, from morning to evening. Furthermore, given the potential association of zinc levels with acne severity, the proportion of patients with more severe disease can be of significance.

Selenium modulates sebum production and exhibits anti‐inflammatory properties. Acne vulgaris therapy often combines vitamin E and zinc due to their antioxidant properties.^3^, ^19^ By comparing female acne patients with a female control group, Moazen et al.^20^ demonstrated lower but not statistically significant selenium levels in acne patients. However, in a systematic review and meta‐analysis, Ai et al.^8^ observed low blood selenium levels in individuals with acne. It is important to note that although serum selenium levels were significantly lower in acne patients of the current study than in controls, the frequency of selenium deficiency was comparable between groups.

Vitamin D is a lipid‐soluble hormone that is obtained through dietary sources and synthesized in the skin upon exposure to sunlight.^21^ Vitamin D exerts its anti‐inflammatory effects through various biological mechanisms. These mechanisms provide evidence for the immune‐regulatory role of vitamin D and its anti‐inflammatory effects in individuals with acne. Vitamin D suppresses the differentiation of T helper 17 (Th17) cells induced by *Cutibacterium acnes*. Reducing interleukin 17 (IL‐17), an inflammatory cytokine, has been observed to be elevated in individuals with acne.^22^ Additional mechanisms induce antimicrobial effects by stimulating the production of antimicrobial peptides, such as cathelicidin, in human sebocytes.^23^ Vitamin D has been found to decrease the production of inflammatory cytokines, including interleukin IL‐6, IL‐8, and matrix metalloproteinase 9, in cultured sebocytes.^24^

Alhetheli et al.^10^ found significantly lower vitamin D levels in patients with acne vulgaris compared to matched healthy controls, which is in line with the current study's results. Nonetheless, contrary to our findings, they observed no correlation between acne severity and vitamin D levels. Also, in contrast to our results, Toossi et al.^25^ found no significant disparity in serum vitamin D levels between individuals with acne and the control group. Additionally, they reported a lack of association between serum vitamin D levels and the severity of the disease. The absence of a correlation between vitamin D levels and acne severity may be attributed to the method used to classify patients in terms of disease severity.

This study's main strength was its larger sample size compared to previous research on patients with acne vulgaris. Nevertheless, there were several limitations. One limitation was that the sample size calculation was only based on serum zinc levels from a previous study, and the power may have not been sufficient for the comparison of serum selenium and vitamin D levels between cases and controls. Another limitation was not considering the dietary status and nutritional intake of the participants. Additionally, the amount of sunlight exposure can influence vitamin D levels, which was not taken into account.

## 5 CONCLUSIONS

Patients diagnosed with acne vulgaris exhibited decreased serum levels of zinc, selenium, and vitamin D in comparison to the control group. Moreover, a relationship existed between disease severity and reduced levels of these nutrients. Future research should take into account various factors that may influence the serum levels of nutrients or the occurrence of acne vulgaris, including nutrition status, sunlight exposure, and hormonal disorders.

## AUTHOR CONTRIBUTIONS

Conceptualization and study validation: Armaghan Kazeminejad, Zohreh Hajheydari, Nasim Gholizadeh. Implementation and supervision: Armaghan Kazeminejad, Zohreh Hajheydari, Seyed Shokoufe Taghian. Data analysis and interpretation: Armaghan Kazeminejad. Writing and reviewing: Nasim Gholizadeh. All authors have read and approved the final version of the manuscript.

## FUNDING INFORMATION

Mazandaran University of Medical Sciences funded the current study.

## CONFLICT OF INTEREST STATEMENT

The authors declare that they have no competing interests.

## ETHICS STATEMENT

The study obtained ethics approval from the Ethics Committee of Mazandaran University of Medical Sciences (ethics code: IR.MAZUMS.REC.1398.1109) and adheres to the principles outlined in the Declaration of Helsinki.

## CONSENT

All participants provided written informed consent.

## Data Availability

The datasets used and/or analyzed during the current study are available from the corresponding author upon reasonable request.

## References

[1] Tan JKL , Bhate K . A global perspective on the epidemiology of acne. Br J Dermatol. 2015;172 Suppl 1:3‐12. 10.1111/bjd.13462 25597339

[2] Bhate K , Williams HC . Epidemiology of acne vulgaris. Br J Dermatol. 2013;168(3):474‐485. 23210645 10.1111/bjd.12149

[3] Podgórska A , Puścion‐Jakubik A , Markiewicz‐Żukowska R , Gromkowska‐Kępka KJ , Socha K . Acne Vulgaris and Intake of Selected Dietary Nutrients—A Summary of Information. Healthcare (Basel, Switzerland). 2021;9(6):668. 34205209 10.3390/healthcare9060668 PMC8226785

[4] Bhate K , Williams HC . What's new in acne? An analysis of systematic reviews published in 2011‐2012. Clin Exp Dermatol. 2014;39(3):273‐277. quiz 7–8, 278. 24635060 10.1111/ced.12270

[5] Ogawa Y , Kinoshita M , Shimada S , Kawamura T . Zinc and skin disorders. Nutrients. 2018;10(2):199. 29439479 10.3390/nu10020199 PMC5852775

[6] Yee BE , Richards P , Sui JY , Marsch AF . Serum zinc levels and efficacy of zinc treatment in acne vulgaris: a systematic review and meta‐analysis. Dermatol Ther. 2020;33(6):e14252. 32860489 10.1111/dth.14252

[7] Cervantes J , Eber AE , Perper M , Nascimento VM , Nouri K , Keri JE . The role of zinc in the treatment of acne: a review of the literature. Dermatol Ther. 2018;31(1):e12576. 10.1111/dth.12576 29193602

[8] Ai P , Lei S , Zhou F , Chen S , Zhang Y . Selenium levels and skin diseases: systematic review and meta‐analysis. J Trace Elem Med Biol. 2020;62:126548. 32497930 10.1016/j.jtemb.2020.126548

[9] Yildizgören MT , Togral AK . Preliminary evidence for vitamin D deficiency in nodulocystic acne. Dermatoendocrinol. 2014;6(1):e983687. 26413187 10.4161/derm.29799 PMC4580068

[10] Alhetheli G , Elneam AIA , Alsenaid A , Al‐Dhubaibi M . Vitamin D levels in patients with and without acne and its relation to acne severity: a case‐control study. Clin Cosmet Investig Dermatol. 2020;13:759‐765. 10.2147/CCID.S271500 PMC7549021 33116739

[11] Saleh BOM , Anbar ZNH , Majid AY . Serum trace elements (zinc, copper and magnesium) status in Iraqi patients with acne vulgaris:(case‐controlled study). Iraqi J Pharm Sci. 2011;20(2):44‐49.

[12] Tutakne M , Chari K . Acne, rosacea, and perioral dermatitis. Dermatology. 2003;2:689‐710.

[13] Zou P , Du Y , Yang C , Cao Y . Trace element zinc and skin disorders. Front Med. 2022;9:1093868. 10.3389/fmed.2022.1093868 PMC9887131 36733937

[14] Butool F , Mohammed A , Syed PA , Mohammed RA . Role of serum zinc and copper levels in patients with acne vulgaris. Journal of orofacial. Research. 2019;8(4):71‐75.

[15] Choi S , Liu X , Pan Z . Zinc deficiency and cellular oxidative stress: prognostic implications in cardiovascular diseases. Acta Pharmacol Sin. 2018;39(7):1120‐1132. 29926844 10.1038/aps.2018.25 PMC6289396

[16] Usmani TM , Alam SM , Ghafoor R , Latif AQ , Saeed F . Association of serum zinc levels with acne vulgaris: a case‐control study. Pak J Health Sci. 2022;3(7):195‐198.

[17] Sultana T , Akter A , Zohra FT , Rahman MQ , Kabir Y . Association of serum zinc and vitamin E levels with acne vulgarisin Bangladeshi acne patients. J Bangladesh Acad Sci. 2021;45(1):49‐58.

[18] Chandra S . Evaluation of serum zinc levels in acne patients: a case‐control study. J Adv Med Dent Scie Res. 2021;9(6):57‐59.

[19] Al‐Anbari HH , Sahib AS , Raghif A . Effects of silymarin, N‐acetylcysteine and selenium in the treatment of papulopustular acne. Oxid Antioxid Med Sci. 2012;1:201‐207.

[20] Moazen M , Mazloom Z , Jowkar F , Nasimi N , Moein Z , Vitamin D . Adiponectin, oxidative stress, lipid profile, and nutrient intakes in the females with acne vulgaris: a case‐control study. Galen Med J. 2019;8:e1515. 34466520 10.31661/gmj.v8i0.1515 PMC8343516

[21] Wacker M , Holick MF . Sunlight and Vitamin D: a global perspective for health. Dermatoendocrinol. 2013;5(1):51‐108. 24494042 10.4161/derm.24494 PMC3897598

[22] Agak GW , Qin M , Nobe J , et al. Propionibacterium acnes induces an IL‐17 response in acne vulgaris that is regulated by vitamin a and vitamin D. J Invest Dermatol. 2014;134(2):366‐373. 23924903 10.1038/jid.2013.334 PMC4084940

[23] Lee WJ , Cha HW , Sohn MY , Lee S‐J , Kim DW . Vitamin D increases expression of cathelicidin in cultured sebocytes. Arch Dermatol Res. 2012;304:627‐632. 22695798 10.1007/s00403-012-1255-z

[24] Lee WJ , Choi YH , Sohn MY , Lee S‐J . Expression of inflammatory biomarkers from cultured sebocytes was influenced by treatment with vitamin D. Indian J Dermatol. 2013;58(4):327. 10.4103/0019-5154.113959 PMC3726901 23919024

[25] Toossi P , Azizian Z , Yavari H , Fakhim TH , Amini SH , Enamzade R . Serum 25‐hydroxy vitamin D levels in patients with acne vulgaris and its association with disease severity. Clin Cases Miner Bone Metab. 2015;12(3):238‐242. 26811702 10.11138/ccmbm/2015.12.3.238 PMC4708967

